# Discrete Survival Model Analysis of *Plasmodium falciparum* Response to Artemisinin-Based Combination Therapies among Children in Regions of Varying Malaria Transmission in Cameroon

**DOI:** 10.3390/pathogens10091106

**Published:** 2021-08-30

**Authors:** Akindeh M. Nji, Innocent M. Ali, Peter Thelma Ngwa Niba, Evehe Marie-Solange, Christian Heumann, Guenter Froeschl, Wilfred F. Mbacham

**Affiliations:** 1 The Biotechnology Center, University of Yaoundé I, Yaoundé BP 8094, Cameroon; akindeh@yahoo.com (A.M.N.); dr.alinn@gmail.com (I.M.A.); thelma2009@yahoo.co.uk (P.T.N.N.); seveheb@yahoo.com (E.M.-S.); 2 Department of Biochemistry, University of Yaoundé I, Yaoundé BP 8094, Cameroon; 3 Center for International Health, Ludwig Maximilian University of Munich, 80802 Munich, Germany; froeschl@lrz.uni-muenchen.de; 4 Department of Biochemistry, University of Dschang, Dschang BP 67, Cameroon; 5 Department of Statistics, Ludwig Maximilian University of Munich, 80802 Munich, Germany; chris@stat.uni-muenchen.de; 6 Division of Infectious Diseases and Tropical Medicine, University Hospital, Ludwig-Maximilians-Universität, 80802 Munich, Germany

**Keywords:** *Plasmodium* *falciparum*, parasite clearance, children, ACT, discrete time, survival model, Cameroon

## Abstract

The need to monitor changes in parasite clearance following treatment with artemisinin-based combination therapies (ACTs) is important in the containment of drug resistance. This study aimed to model *Plasmodium falciparum* response to ACTs among children in two different transmission settings (Mutengene and Garoua) in Cameroon. Using the step function, a discrete-time survival model was fitted with all the covariates included that might play a role in parasite clearance. The probability of clearing parasites within 24 h following treatment was 21.6% and 70.3% for younger children aged 6 to 59 months and 29.3% and 59.8% for older children aged 60 to 120 months in Mutengene and Garoua, respectively. After two days of treatment, the conditional probability of clearing parasites given that they were not cleared on day 1 was 76.7% and 96.6% for children aged 6–59 months and 83.1% and 93.5% for children aged 60–120 months in Mutengene and Garoua, respectively. The model demonstrated that the ecological setting, age group and pretreatment serum levels of creatinine and alanine aminotransferase were the main factors that significantly influenced parasite clearance in vivo after administration of ACTs (*p* < 0.05). The findings highlight the need for further investigations on host differential response to ACTs in current practice.

## 1. Introduction

The introduction of highly efficacious artemisinin-based combination treatments (ACTs) as first-line treatment in most malaria-endemic countries has recently contributed to notable reductions in childhood morbidity and mortality across sub-Saharan Africa [1,2]. However, clinical and molecular studies suggest de novo emergence of artemisinin-resistant *Plasmodium falciparum* parasites in the Thailand–Cambodia border area, where the standard ACT is artesunate–mefloquine [3,4]. The *P*. *falciparum* Kelch 13 (*K13*) propeller variants are the main markers responsible for resistance to artemisinins [5]. In Africa, even though resistance to the artemisinins is not yet a threat to malaria treatment, recent studies have reported the presence of R561H and P574L polymorphisms conferring partial resistance to ACTs [6,7,8,9]. The human host factors responsible for delayed parasite clearance after the administration of ACTs have not been fully understood. By measuring parasite clearance rates in genetically identical and nonidentical parasites, some authors found that variations in parasite clearance following treatment with artemisinin derivatives were largely affected by parasite genetic elements [10]. Drug pressure in communities where self-medication is common has the potential of reducing parasite sensitivity to drugs, resulting in the selection of resistant clones [11,12]. Furthermore, factors such as endemicity and host immunity play a crucial role in defining the emergence and spread of drug resistance [13,14].

Using data from different studies, a few models have been fitted to describe patterns in antimalarial drug resistance and efficacy. Some of these models have described patterns in drug use, strategies to delay the progress of drug resistance [15], the role of antimalarial drugs in elimination of malaria [16] and the impact of ACTs and long-acting treatments in reducing malaria transmission [17]. Pongtavornpinyo and colleagues in 2008 described several factors that may affect ACT efficacy [18]. Treatment failure, coverage with ACTs, self-medication and presumptive treatment were identified as affecting resistance depending on the transmission level. This model measured the spread of drug resistance with the assumption that it already existed. Furthermore, Okell and colleagues in modeling intrahost parasite dynamics treated with ACTs demonstrated that parasite ring stage specificity to drug sensitivity was driving artemisinin resistance [17].

Although there are artemisinin resistance containment strategies spearheaded by the World Health Organization (WHO), combating this resistance will require many unknowns to be addressed [19]. Indeed, Maude et al. uncovered parasite clearance half-life as a strong predictor of the likelihood of artemisinin resistance individual patients and suggested that the “day 3” threshold of 10% parasitemia reduction might be a relevant measure to predict the likelihood of resistance development [16]. However, this result is relevant in low transmission settings [16]. Therefore, in modeling the ACT response in humans treated for uncomplicated *P*. *falciparum* malaria, the incorporation of ecological and parasite factors has the potential to uncover other likely predictors of artemisinin resistance, especially in high transmission settings in Africa where the emergence of artemisinin resistance is yet to occur. The time to parasite clearance with respect to these factors could also be helpful in understanding the progress of artemisinin resistance.

## 2. Materials and Methods

### 2.1. Study Design

Data from a three-arm, open-labeled, randomized controlled noninferiority trial comparing the efficacy, safety and tolerability of artesunate–amodiaquine (AS-AQ) and dihydroartemisinin–piperaquine (DHA-PPQ) to artemether–lumefantrine (AL) in children aged 6 to 120 months during a 42-day follow-up period were used [20]. 

### 2.2. Study Site and Setting

Participants were enrolled from two ecological regions in Cameroon with different malaria transmission patterns. Mutengene, a malaria holoendemic junction town that is in the semi-mangrove and tropical wet forest region, has perennial transmission with seasonal spikes, while Garoua, which is in the Guinea Sahel–savannah belt, experiences seasonal malaria with marked spikes in transmission during the beginning of the rainy season in May–June.

### 2.3. Study Population

Participants in this study were *P*. *falciparum* malaria positive patients, either gender and 6 to 120 months of age visiting the hospital for care. 

Participants were enrolled if (i) they were microscopically confirmed (using Giemsa-stained thick film) with uncomplicated *P. falciparum* malaria with 1000–100,000 asexual parasites/μL, (ii) they had fever with an axillary temperature ≥37.5 °C or history of fever in the past 24 h, (iii) they were able to ingest tablets orally and (iv) their parents or guardians gave consent on their behalf to take part in the study and visit the clinic on days of follow-up.

Participants were excluded if (i) they had mixed infection with other *Plasmodium* species, (ii) they were microscopically confirmed (using Giemsa-stained thick film) with uncomplicated *P. falciparum* malaria with <1000 or >100,000 asexual parasites/μL, (iii) they had an absence of fever or an axillary temperature <37.5 °C, (iv) they had any danger signs of severe malaria, (v) they were unable to ingest tablets orally or (vi) their parents or guardians refused to give consent on their behalf to take part in the study and visit the clinic on days of follow-up.

### 2.4. Study Procedure

Giemsa-stained thick and thin blood smears were prepared from finger-pricked blood samples according to WHO guidelines to assess the presence and quantification of malaria parasites [21,22]. Primary inclusion into the study was based on a positive thick smear reading. Blood smears were examined under a microscope for determination of parasitemia on all visit days (days 0, 1, 2, 3, 7, 14, 21, 28, 35 and 42) and for all unscheduled visits. Using the same clean microscope slide, approximately 5 μL of capillary blood was used for thick smear preparation, and 2 μL was used for thin smear preparation. The smears were allowed to air dry and the thin smears were fixed in absolute methanol before staining with freshly prepared 10% Giemsa stain for 15–20 min [22]. The slides were washed with tap water and air-dried before examination using 100× objective light microscope [22]. Parasitemia was quantified by a standard approximation method (40 × the number of parasites per 200 leucocytes on thick film) [22]. Malaria parasite speciation and detection of gametocytes (asexual stages) were done using the thin blood films [22]. The gametocytes were reported as present or absent. Two trained microscopists observed the slides [22]. Quality control of the microscopy readings was done by mask reading of 10% of the slides at the Immunology Laboratory of the Biotechnology Center, University of Yaoundé I. The average parasitemia was calculated in cases with discordant readings of less than 50% between the first two readers. However, the expert microscopist was used when the readings had a disagreement of more than 50% between the first and second readers [22]. 

Hemoglobin levels were measured on days 0, 7 and 42 and on any unscheduled visit using a HemoCue B-Hemoglobinometer (HemoCue, Ängelholm, Sweden). A full blood count, including differentials, and biochemical parameters of liver and kidney functions (alanine aminotransferase serum activity, bilirubin and creatinine serum concentrations), were investigated from venous blood before treatment and on days 7 and 42 or on the day of reappearance of parasitemia.

Polymerase chain reaction (PCR) analysis of the polymorphic antigen markers merozoite surface proteins (*msp-1*, *msp-2*) and glutamate-rich protein (*glurp*) of *P*. *falciparum* parasites was used to distinguish reinfections from recrudescences in all parasite recurrences during the period of follow-up. Malaria cases were classified as new infections when there were no common bands observed between day 0 and the day of recurrent parasitemia. However, a case was categorized as recrudescence when there was at least one common band between baseline sample and that of the day of parasite reappearance for any of the 3 markers (even if there were additional bands on day 0). Malaria parasite infected cases were considered not to be clinical failures if their recurrent parasitemia were classified as new infections rather than recrudescent infections. There were 720 children recruited in this study. The results of the randomized three-arm, open-label, noninferiority clinical trial were published elsewhere [20].

### 2.5. Statistical Analysis

Parasite density for each patient was determined in the morning on the fixed visit days. Therefore, only the interval of time when parasites were cleared for each patient was known. Hence, a discrete-time survival model was fitted instead of the conventional proportional hazard model which assumes time to event to be continuous and that the exact instance of parasite clearance is known. Any visit in which the patient’s parasitemia was microscopically undetectable was considered an event. Those who had cleared their parasites before being lost to follow-up and those not lost to follow-up who did not clear their parasites were included in the data set. The covariates used in the model were as follows: site (Garoua or Mutengene), the administered drug (AL, DHA-PPQ or AS-AQ), the residence type (urban or rural), the sex (male or female), age group (6 to 59 months, 60 to 120 months), weight and pretreatment temperature. Possible interacting factors were simultaneously included in the model to assess their combinatorial effect on parasite clearance in vivo. The combinations included the following: age and drug, site and drug. Given that some liver and kidney function tests and hematological parameters were assessed only on days 0, 7 and 42, only the pretreatment values were considered with the assumption that their correlation to parasite clearance would be most relevant before treatment. To this end, the following parameters were also included in the model: alanine aminotransferase (ALAT) levels (normal range: 3–61 U/L), creatinine levels (normal range: 0.5–1.4 mg/dL), relative neutrophil levels (as per proportion of leucocyte count; normal range: 42–72%) and hemoglobin levels (normal range: ≥10 g/dL). The liver and kidney function tests were dichotomized in the model. The acetylator status (slow acetylator and fast acetylator) was also included as a predictor in the model. The phenotype of drug metabolic status was determined using polymerase chain reaction–restrictive fragment length polymorphism (PCR-RFLP) of the phase II enzyme encoded by the N-acetyltransferase 2 (*NAT2*) gene. This approach was used as a proxy for the measurement of pharmacokinetics in the absence of blood drug concentration.

A discrete-time survival model was fitted with day of visit as the discrete time given that only the interval was recorded rather than using the precise hour during which the parasites would no longer be detectable microscopically. The time points were the days patients visited the clinic for clinical evaluation. The hazard function was obtained by reparameterizing these probabilities to have a logistic dependence on predictors and time. The discrete hazard model is given by:

(1) $$h_{ij}=\frac{1}{{}^{1+\exp(-[(\alpha_{{}^{1}}D_{1\mathrm{ij}}+\alpha_{2}D_{2\mathrm{ij}}+\ldots +\alpha_{J}D_{\mathrm{Jij}})+(\beta_{{}^{1}}Z_{1\mathrm{ij}}+\beta_{2}z_{2\mathrm{ij}}+\ldots +\beta_{P}Z_{\mathrm{Pij}})])}}$$

where $h_{ij}$ is the probability that an individual i experiences the event at time j and [D_1ij_, D_2ij_, …, D_Jij_] are a sequence of dummy variables with values [d_(1)ij_, d_(2)ij_, …, d_(J)ij_] indexing time periods. In this model, “J” refers to the day of clearance, [α_1_, α_2_, …, α_J_] represent the baseline level of hazard in each time period and the slopes [β_1_, β_2_, …, β_p_] describe the effects of predictors on the baseline hazard function on a logistic scale. The following equation was obtained by taking the logit transformation of Equation (1) above [23]:

(2) $$\log_{e}(\frac{h_{ij}}{1-h_{ij}})=(\alpha_{{}^{1}}D_{1\mathrm{ij}}+\alpha_{2}D_{2\mathrm{ij}}+\ldots +\alpha_{J}D_{J\mathrm{ij}})+(\beta_{{}^{1}}Z_{1\mathrm{ij}}+\beta_{2}z_{2\mathrm{ij}}+\ldots +\beta_{P}Z_{\mathrm{Pij}})$$

Hazard functions transformed this way provided the conditional log odds that an event will occur in each time point (visit day) given that the individual did not experience the event in the previous time point (visit). This is expressed as a linear function of α_j_ specific to time j, with the values of the predictors at time j multiplied by the appropriate slopes (β_j_).

This model was fitted using “*glm*” function in R statistical software version 4.1.0 (supported by the *R* Foundation for Statistical Computing, Vienna, Austria) with logit function as link function. To fit this model using logistic regression, the data were first converted to a person-time data set such that each participant had one record corresponding to the discrete observed time. Therefore, the number of records for each participant corresponded to the number of discrete observed times until the event was observed (Supplementary Table S1).

The hazard conditional probabilities were assessed by plotting predicted probabilities per time point and per predictor. Based on the fitted conditional hazard probabilities, the survival probability at time j can be obtained by Equation (3):

(3) $${\hat{s}}_{j}=\overset{k}{\underset{k=1}{\prod }}(1-{\hat{h}}_{k})$$

where ${\hat{h}}_{k}$ is the conditional probability at time *k*. The estimated values of *S_j_* where *j* = 1, 2, …, m were used to plot the survival function.

The discrete-time model was fitted on the assumption that the linear–logistic model was a valid representation of reality (linearity) and that all heterogeneity across individuals was accounted for by the variation of the values of the covariates. It was also assumed that the logit-hazard profiles corresponding to all possible values of every predictor are distinguished only by their constant vertical separation (proportionality). Linearity assumption was confirmed by fitting a generalized additive model while smoothing all the continuous variables. The degrees of freedom (df) of the continuous variables close to 1 indicated that the linearity assumption was tenable. To investigate the assumption of no unobserved heterogeneity, a mixed model was fitted with each subject as a random effect. A very small variation accounted for by the random effect showed that all the covariates in our model covered almost all the variation in the data. The proportionality assumption was investigated by verifying that the logit-hazard profiles estimated separately within strata were all approximately parallel.

In all analyses, two-tailed *p* values less than 0.05 at 95% confidence level were considered to be statistically significant.

## 3. Results

### 3.1. Description of the Study Participants and Parameters 

In this study, participants were randomized to receive three ACTs at Mutengene (AS-AQ = 144, DHA-PPQ = 144 and AL = 72) and Garoua (AS-AQ = 144, DHA-PPQ = 144 and AL = 72), which are ecologically different. Out of 720 enrolled patients, 23 (3.2%) dropped out after the first dose, 74 (10.3%) were lost to follow-up prior to reaching parasite clearance and 623 (86.5%) were used in the modeling phase. This study included the following parameters: site, drug type, age, ecotype, urbanicity, parasite density, weight, temperature, hemoglobin level, relative neutrophil level, drug metabolizing status, alanine aminotransferase level and creatinine level.

### 3.2. Association between Study Sites and Parasite Density

There was a difference in the geometric mean parasite count between the two sites (31,039 asexual parasites/μL for Garoua and 33,479 asexual parasites/μL for Mutengene). However, the mean log parasite counts for the two sites were not significantly different (*t* = −1.576, degree of freedom (df) = 613, *p* = 0.11). The 95% confidence interval (95% CI) of the log parasite count was −0.15 to 0.02, indicating that between sites, there was no significant difference (*p* > 0.05) in pretreatment parasite density.

### 3.3. Discrete-time Survival Model Diagnostics

The degrees of freedom (df) greater than 1 in a generalized additive model involving smoothened continuous variables are indicative of a breakdown in the linearity assumption. Hence, a generalized additive model was fitted using the variables of the full model and smoothing the continuous variables (weight, pretreatment temperature) in the model. The variable “weight” did not show any challenge to the assumption of linearity (degree of freedom of 1.001). However, the variable “temperature” showed a deviation from the linearity assumption (degree of freedom of 3.077), which was not statistically significant (*p* = 0.16). Therefore, the linearity assumption of the predictors was tenable.

Fitting a random intercept model to the final selected predictors, with subjects or individual patients as random effects, we found the variance captured by random effects to be 7.8 × 10^−7^ (standard deviation of 2.8 × 10^−4^). The magnitude of variance can be considered negligible. Therefore, the assumption of unobserved heterogeneity uncorrelated with the independent variables in the model was valid. Separate plots of logit hazard functions within each stratum were approximately parallel. Proportionality was therefore assumed. Hence, there is evidence that the assumptions for this discrete-time survival model were reasonable.

### 3.4. Survival Model Fitting

The model arrived at was a discrete-time survival model incorporating time and all variables suspected of influencing parasite clearance (Table 1). The model showed that significant parasite clearance rates occurred on days 1, 2, 3, 7 and 14 (*p* < 0.05). Similarly, the site (Mutengene) and normal serum ALAT level on day 0 demonstrated a significant effect on parasite clearance (*p* < 0.05). Conversely, normal pretreatment serum creatinine level was significantly associated with delay in parasite clearance (*p* < 0.05). However, the other predictors did not show any significant effect (*p* > 0.05). The interaction between age group and site was important in determining time to parasite clearance at a 5% significance level. 

The odds ratios of variables in the final model showed that those with normal pretreatment ALAT activity (normal (3–61 U/L)) had a higher probability of clearing their parasites earlier (OR = 1.58, 95% CI: 1.15–2.01) when compared to those with abnormal levels. However, having normal creatinine (normal (0.5–1.4 mg/dL)) levels was associated with delay in parasite clearance (OR = 0.53, 95% CI: 0.12–0.67) compared to those having abnormal creatinine levels (less than the lower normal limit—<0.5 mg/dL). Parasite clearance appeared to be delayed in Mutengene (OR = 0.12, 95% CI: 0.07–0.18) when compared to Garoua. In Mutengene, older children (60–120 months) had a higher probability (OR = 2.72, 95% CI: 2.11–3.33) than children aged 6–59 months of clearing their parasites (Table 2).

**Figure 1:**
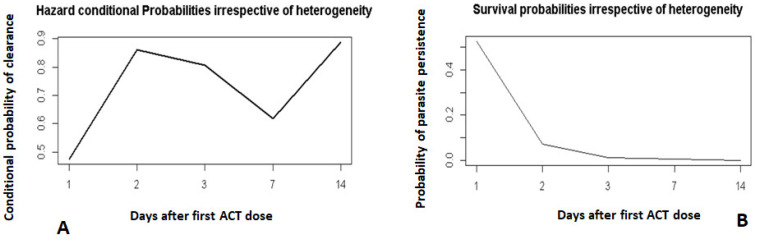
Hazard and survival probability profiles of the time effect on parasite clearance. Hazard conditional probabilities irrespective of heterogeneity (**A**). Survival probabilities irrespective of heterogeneity (**B**).

The time effect on the hazard function (Figure 1A) showed that on day 2, there was a higher probability of children clearing their parasites if they had not already done so on day 1. By day 3, most children had cleared their parasites (Figure 1B).

Day 0 was not included. It corresponds to the day when the first dose of ACT was administered.

**Figure 2:**
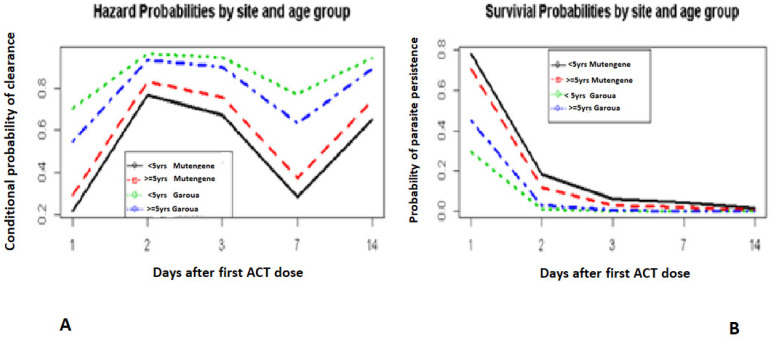
Hazard and survival probabilities by site and age group. Hazard probabilities by site and age group (**A**). Survivial probabilities by site and age group (**B**).

Children in Mutengene lagged behind those in Garoua in parasite clearance, especially in the first two days after treatment (Figure 2A,B). Older children in Mutengene (60–120 months; day 1: 29.3%, day 2: 83.1%) had a better parasite clearance prognosis compared to the younger ones (6–59 months; day 1: 21.6%, day 2: 76.6%) (Figure 2A). In Garoua (Figure 2B), the opposite trend was observed.

Day 0 was not included. It corresponds to the day when the first dose of ACT was administered.

**Figure 3:**
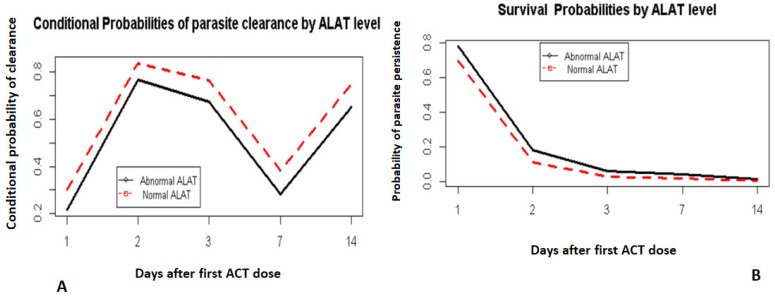
Hazard and survival probabilities by ALAT level. Conditional probabilities of parasite clearance by ALAT level (**A**). Survival probabilities by ALAT level (**B**).

The ALAT levels in patients before treatment were correlated with time to parasite clearance (Figure 3A,B). Those with normal ALAT levels before treatment remained consistent with a higher chance of clearing their parasites for all the visit days on the condition that they did not clear the previous day of visit (Figure 3A). The probabilities of clearing parasites 2 days after treatment given that they were not cleared 1 day after treatment for normal and abnormal ALAT levels were 83% and 76%, respectively (Figure 3A).

Day 0 was not included. It corresponds to the day when the first dose of ACT was administered.

**Figure 4:**
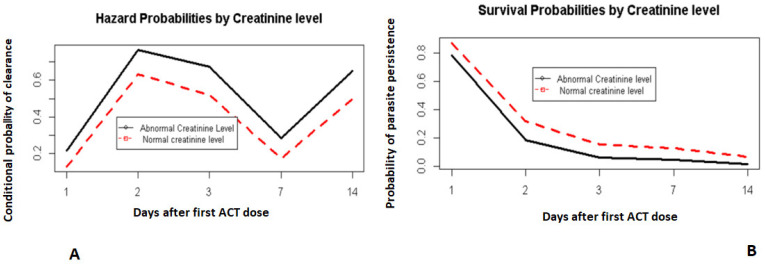
Hazard and survival probabilities by creatinine levels. Hazard probabilities by creatinine level (**A**). Survival probabilities by creatinine level (**B**).

Lower time-to-clearance was also correlated in part with the participants’ creatinine levels before treatment (Figure 4A,B). Those with a normal creatinine level (within normal limits) had a lower chance of clearing parasites by each visit day given they had not cleared in the previous day than those with abnormal creatinine levels (below the normal limits). 

Day 0 was not included. It corresponds to the day when the first dose of ACT was administered.

**Figure 5:**
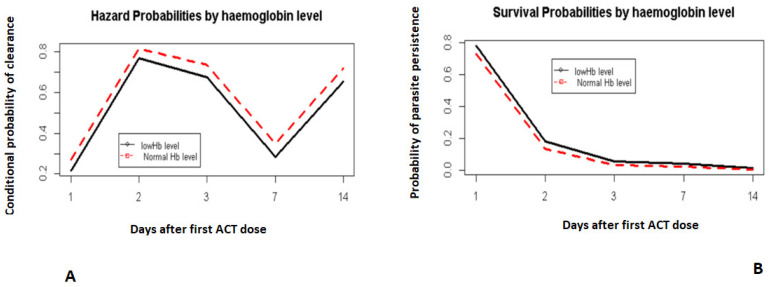
Hazard and survival probabilities by hemoglobin. Hazard probabilities by haemoglobin level (**A**). Survival probabilities by haemoglobin level (**B**).

Although hemoglobin levels were not strongly correlated with parasite clearance, there was a trend in which those with lower pretreatment hemoglobin levels appeared to lag behind those with a normal pretreatment hemoglobin level (Figure 5A,B). This relationship was statistically insignificant (*p* > 0.05). 

Generally, there was no significant difference (*p* > 0.05) in the parasite clearance rates with time when comparing patients on the three test drug regimens.

Day 0 was not included. It corresponds to the day when the first dose of ACT was administered.

## 4. Discussion

This study aimed to model *P*. *falciparum* clearance among children aged 6 to 120 months in two ecologically different regions (Mutengene and Garoua) following treatment with AL, AS-AQ and DHA-PPQ. A discrete-time survival model was fitted to predict the factors in the two different transmission settings that may be correlated with parasite clearance during the early days of treatment. 

There are many contributing and interrelated factors that may be associated with host response to parasite clearance [24]. Integrating these factors in models gives a better estimation of the extent to which each contributing factor is changing the rate of parasite clearance. In the present study, the interaction between age group and site was significantly associated with parasite clearance. In Mutengene, older children (60–120 months) had a higher probability of clearing the parasites when compared to the younger children (6–59 months). This observation is consistent with reports that immunity to malaria is age-dependent [25]. In a study that modeled factors associated with the half-life following treatment with ACT, the authors observed that clearance of parasites irrespective of artemisinin treatment was related to immunoglobulins directed against erythrocyte surface ligands such as *Plasmodium falciparum* erythrocyte membrane protein 1 (*PfEMP1*) [26]. This effect was plasma-level-dependent and increased with age [26]. Contrarily, different studies carried out in Mozambique [27] and Malawi [28] identified the age of first exposure to *P*. *falciparum* as an insignificant predictor of antibody acquisition. In Garoua, a region with low humidity, it was observed that younger children appeared to clear parasites faster than older children. This observation is inconsistent with findings from an area with similar ecological endemicity and characteristics [26]. Our finding appeared to disagree with those of a study conducted in a low malaria transmission area in Mali that reported increase in age as a surrogate marker for immunity [26]. Previous studies have documented lower susceptibility to malaria infections in sympatric ethnic populations, and this was associated with higher malaria-specific immunoglobulin plasma levels, especially among the Fulani [29,30,31]. 

The model indicated that children in Mutengene lagged behind those in Garoua in clearing their parasites, especially during the first two days after drug administration. While the efficacies of the ACT were high and comparable [20], independent risk factors that accounted for persistent parasitemia on days 1 and 2 after drug administration included endemicity and age. An opposite age effect was seen between sites, which may be due to differences in chronologies in the development of malaria immunity. On the other hand, pretreatment hemoglobin level had a moderate effect on parasite persistence, although not significant at the 5% significance level.

Patients with normal pretreatment serum ALAT activity and abnormal pretreatment serum creatinine levels appeared to clear their parasites faster than those with abnormal levels. Conversely, it was observed that children with normal creatinine levels cleared parasitemia more slowly after treatment. Abnormal ALAT activity and creatinine levels may be indicative of liver and kidney injuries, respectively. This may lead to compromises in the ability of patients to adequately maintain efficacious clearance patterns since increasingly resistant parasite forms may multiply in the presence of low drug concentrations. Self-medication is a common practice in most patients living in rural areas prior to hospital visit. The combined effect of the infection and self-medication could also increase the risk of not clearing parasites efficiently. Moreover, it was noticed that the rates of clearance between age groups and sites were not significantly different by day 3. None of these parameters was associated with treatment failure during the study period, and efficacy levels remained high [20]. 

It has recently been shown through haplotype analysis of well-characterized clinical isolates of *Plasmodium falciparum* that genetic resistance to the artemisinins is mediated by polymorphisms in the gene encoding the Kelch propeller domain 13, and these mutations arise independently in several locations [32]. Studies conducted in Tanzania and Rwanda have also associated the nonsynonymous *P*. *falciparum* Kelch 13 mutations (P574L and R561H) with delayed parasite clearance [6,7,8,9]. The implication of these observations in close monitoring of resistant parasites cannot be overestimated especially in endemic areas where case management relies principally on the use of ACTs.

The major strength of this study is that it reported for the first time the factors influencing in vivo malaria parasite clearance in response to AS-AQ, AL and DHA-PPQ in two main transmission settings in Cameroon. The findings from this study are still valid today despite the fact that the clinical data were obtained from studies conducted between 2009 and 2013. This is because no recent data have shown a change in malaria transmission patterns in Mutengene and Garoua. Moreover, the ACTs are currently used in these areas in the treatment of uncomplicated *P*. *falciparum* malaria. Furthermore, results from this study add to baseline knowledge for ongoing investigations into regional differences in response to malaria interventions.

The limitations of this study are as follows: (1) The sample schedule for parasite density determination to estimate rates of parasite clearance was not optimal. Measurements using shorter time intervals are recommended by WHO in the early clearance phase after treatment initiation. With limited resources, the only operationally feasible strategy was to measure parasites on patient visit days. (2) Clearance times were estimated and not rates, with the latter more generally accepted to be a better measure of clearance. However, fitting parasite clearance in our discrete survival model provided an alternative way of effectively studying the behavior of parasites in the presence of drugs in sites of different malaria endemicities early during treatment. The plasma drug levels could not be measured to rule out reduced adherence or impairment in drug metabolism as causes of differences observed among study participants. Even though acetylation was used as a proxy of pharmacokinetics, it does not provide information on plasma drug levels. However, it is worth noting that the ethnic composition is different between the two sites despite the absence of data on ethnicity.

## 5. Conclusions

The model adopted for this study showed a delay in parasite clearance in Mutengene compared to Garoua among children aged 6 to 120 months treated with AS-AQ, DHA-PPQ and AL. The site of study (Mutengene), increasing age (Mutengene) and normal pretreatment creatinine serum levels and normal pretreatment alanine aminotransferase serum levels were the main factors that significantly influenced parasite clearance in vivo after administration of ACTs. Although no differences were seen with parasite clearance on day 3, the observations flag the need for close monitoring of host differential response to artemisinins in Cameroon.

## Figures and Tables

**Table 1:** Discrete-time survival model parameter estimates with standard errors.

Predictors	Model Parameters β (Standard Error)
Day 1	4.63 (0.43) ***
Day 2	7.14 (0.48) ***
Day 3	6.75 (0.65) ***
Day 7	5.04 (0.63) ***
Day 14	6.60 (1.16) ***
Day 21	−9.77 (533.41)
Sex (male)	−0.10 (0.15)
Age group > 5 years	−0.76 (0.40)
Site (Mutengene)	4.05 (1.29) **
AS-AQ	−0.14 (0.36)
DHA-PPQ	−0.17 (0.36)
Fast metabolizer	0.03 (0.16)
Normal neutrophils level day 0 (%)	−0.13 (0.16)
Normal ALAT level day 0 (U/L)	0.46 (0.22) *
Ecotype (Forest)	1.49 (1.23)
Ecotype (Sahel)	−1.26 (1.70)
Urbanicity (Urban)	−0.03 (0.26)
Weight (kg)	0.01 (0.02)
Temperature (°C)	−0.09 (0.11)
Hemoglobin level > 10 g/dL	0.26 (0.16)
Normal creatinine level at day 0 (mg/dL)	−0.64 (0.21) **
Age group > 5 years × AS-AQ	0.12 (0.41)
Age group > 5 years × DHA-PPQ	−0.01 (0.41)
Site (Mutengene) × AS-AQ	0.36 (0.41)
Site (Mutengene) × DHA-PPQ	0.69 (0.42)
Age group > 5 years × Site (Mutengene)	1.00 (0.31) **

Legend: ***, ** and * were statistically significant at *p* < 0.001, *p* < 0.01 and *p* < 0.05, respectively, AS-AQ = artesunate-amodiaquine, DHA-PPQ = dihydroartemisinin–piperaquine, ALAT = alanine aminotransferase.

**Table 2:** Odds ratios and 95% confidence interval for significant variables of the model.

Predictors	Odd Ratio Estimate	95% Confidence Interval
Site (Mutengene)	0.12	(0.07, 0.18) *
Normal ALAT level day 0	1.58	(1.15, 2.01) *
Normal creatinine level at day 0	0.53	(0.12, 0.67) *
Age group > 5 years × Site (Mutengene)	2.72	(2.11, 3.33) *

Legend: * significant predictors, hazard = clearing parasite, ALAT = alanine aminotransferase, normal ALAT level = 3–61 U/L, normal creatinine level = 0.5–1.4 mg/dL.

## Data Availability

All relevant data are provided within the manuscripts. Raw data can be made available upon reasonable request.

## Supplementary Materials

The following are available online at https://www.mdpi.com/article/10.3390/pathogens10091106/s1, Supplementary Table S1. An example of data transformed to person-time data for fitting discrete-time survival model using logistic regression.

## Abbreviations

ACT	Artemisinin-based combination therapy
ALAT	Alanine aminotransferase
AL	Artemether–lumefantrine
AS-AQ	Artesunate–amodiaquine
DHA-PPQ	Dihydroartemisinin–piperaquine
WHO	World Health Organization
