# Supplementary material for: Discrete Survival Model Analysis of Plasmodium falciparum Response to Artemisinin-Based Combination Therapies among Children in Regions of Varying Malaria Transmission in Cameroon
Source: Pathogens. 2021 Aug 30;10(9):1106. doi: 10.3390/pathogens10091106 (PMC8465257; doi:10.3390/pathogens10091106)
Supplement: Supplementary file 1 [file pathogens-10-01106-s001.zip › pathogens-1313011-supplementary.pdf]

Supplementary Table S1. An example of data transformed to person-data time for fitting discrete time survival model using logistic regression

Patient ID	Site code	Drug	Age group	Temp (ºC)	Par (/µl)	Cleared	Visit day
3	1	ASAQ	0	38	52,400	0	0
3	1	ASAQ	0	36	240	0	1
3	1	ASAQ	0	36	0	1	2
4	1	AL	0	38	14,320	0	0
4	1	AL	0	37	160	0	1
4	1	AL	0	37	0	1	2

Legend: "cleared" is the event status variable and "visit day" is the observed discrete time
